# The Effects of Intraoperative Inspired Oxygen Fraction on Postoperative Pulmonary Parameters in Patients with General Anesthesia: A Systemic Review and Meta-Analysis

**DOI:** 10.3390/jcm8050583

**Published:** 2019-04-28

**Authors:** Chang-Hoon Koo, Eun Young Park, Sun Young Lee, Jung-Hee Ryu

**Affiliations:** 1 Department of Anesthesiology & Pain medicine, Seoul National University College of Medicine, Seoul 03080, Korea; vollock9@gmail.com; 2 Department of Anesthesiology & Pain medicine, Seoul National University Bundang Hospital, Seongnam 13620, Korea; 3 Department of Anesthesiology & Pain medicine, Hallym University Sacred Heart Hospital, Hallym University College of Medicine, Anyang 14068, Korea; parkey00@hallym.or.kr; 4 Department of Anesthesiology & Pain medicine, CHA Bundang Medical Center, CHA University School of Medicine, Seongnam 13496, Korea; syhahaha87@naver.com

**Keywords:** oxygen, postoperative complications, risk factors

## Abstract

High intraoperative inspired oxygen concentration is applied to prevent desaturation during induction and recovery of anesthesia. However, high oxygen concentration may lead to postoperative pulmonary complications. The purpose of this study is to compare the postoperative pulmonary parameters according to intraoperative inspired oxygen fraction in patients undergoing general anesthesia. We identified all randomized controlled trials investigating postoperative differences in arterial gas exchange according to intraoperative fraction of inspired oxygen (FiO_2_). A total of 10 randomized controlled trials were included, and 787 patients were analyzed. Postoperative PaO_2_ was lower in the high FiO_2_ group compared with the low FiO_2_ group (mean difference (MD) −4.97 mmHg, 95% CI −8.21 to −1.72, *p* = 0.003). Postoperative alveolar-arterial oxygen gradient (AaDO_2_) was higher (MD 3.42 mmHg, 95% CI 0.95 to 5.89, *p* = 0.007) and the extent of atelectasis was more severe (MD 2.04%, 95% CI 0.14 to 3.94, *p* = 0.04) in high intraoperative FiO_2_ group compared with low FiO_2_ group. However, postoperative SpO_2_ was comparable between the two groups. The results of this meta-analysis suggest that high inspired oxygen fraction during anesthesia may impair postoperative pulmonary parameters. Cautious approach in intraoperative inspired oxygen fraction is required for patients susceptible to postoperative pulmonary complications.

## 1. Introduction

Postoperative pulmonary complications such as atelectasis, respiratory failure and pneumonia have been known to occur in 1–23% of patients undergoing surgery [1]. Risk factors of postoperative pulmonary complications consist of patient factors, surgery type and anesthetic management [1]. Among them, anesthetic management, such as intraoperative ventilation strategy and neuromuscular blockade, affects the pathophysiology of postoperative pulmonary complications after general anesthesia. Most anesthesiologists apply high inspired oxygen fraction during induction and recovery of anesthesia [2]. Preoxygenation during induction of anesthesia could prevent desaturation in the case of airway loss or ventilation failure [2]. High inspired oxygen fraction improves tissue perfusion and wound healing [3,4], but it may generate reactive oxygen species leading to oxidative stress and deoxyribonucleic acid (DNA) damage [5]. In addition, it promotes coronary vasoconstriction, increases peripheral vascular resistance, and decreases cardiac output [6]. 

High intraoperative inspired oxygen concentration—even in the short term—clearly leads to atelectasis formation during general anesthesia [2]. Atelectasis is also one of the most commonly occurring pulmonary complications after general anesthesia, with the incidence of up to 80–90% on the first day after surgery [7], which may still remain until postoperative 4 days [8]. Atelectasis may lead to shunt, which may deteriorate gas exchange and induce hypoxia [9,10]. Additionally, postoperative atelectasis may be associated with morbidities such as pneumonia [11]. Prevention of atelectasis, therefore, could enhance the quality of recovery and patient satisfaction. There are two major mechanisms of atelectasis formation, compression and absorption [12]. Compressive atelectasis is formed by diaphragm displacement, which is common in abdominal surgery, whereas absorptive atelectasis occurs with high inspired oxygen concentration.

Whether intraoperative inspired oxygen fraction affects pulmonary gas exchange after surgery remains controversial. The purpose of this meta-analysis was to compare postoperative pulmonary parameters, including pulmonary gas exchange and atelectasis, according to intraoperative inspired oxygen fraction therapy in patients undergoing general anesthesia.

## 2. Methods

This meta-analysis was conducted according to a Preferred Reporting Items for Systemic Reviews and Meta-Analyses (PRISMA) statement guidelines [13] and the protocol was registered with the International Prospective Register of Systematic Reviews (PROSPERO) (registration no.: CRD 42018117784).

### 2.1. Data Sources and Search Strategy (Literature Search)

We included randomized controlled clinical trials (RCTs) investigating postoperative pulmonary complications according to intraoperative inspired oxygen concentration after surgery. Two authors (C.-H.K., J.-H.R.) independently searched MEDLINE, EMBASE, CENTRAL, CINAHL, Scopus and Web of Science. The last search was performed on August 2018 and relevant studies were retrieved. On search, there were no restrictions regarding publication year and language.

A detailed search strategy for each database is represented in Table S1. It was established with the medical subject headings (MeSH), text words and controlled vocabulary terms and each result was combined by the Boolean operator “AND” or “OR”.

### 2.2. Study Selection

Two authors (C.-H.K., J.-H.R.) independently screened titles and abstracts to select relevant studies from the literature search. Subsequently, full-text of articles was evaluated to include trials which met the eligibility criteria of our review. Any disagreement in the study selection was resolved by discussion and consultation with the third author (S.Y.L.).

### 2.3. Data Extraction and Collection

Two authors (C.-H.K., E.Y.P.) independently extracted and collected data including trials-related data (first author, publication year, language, sample size, design), demographic data (age, anesthetics, type of surgery), intervention-related data (fraction of inspired oxygen, airway device, intervention period) and outcomes (partial pressure of oxygen, alveolar-arterial oxygen gradient, blood oxygen saturation, atelectasis severity). If results were represented only in forms of graph, Engauge Digitizer 10.9 (M. Mitchell, Engauge Digitizer, http://digitizer.sourceforge.net) was used to extract mean and standard deviation (SD). If any discrepancies were found, a third author (S.Y.L.) was consulted. 

### 2.4. Methodological Quality and Risk of Bias Assessment

The Cochrane Risk of Bias tool was used to evaluate methodological quality and risk of bias by two independent authors (C.-H.K., E.Y.P.) [14]. It has seven domains: Random sequence generation, Allocation concealment, Blinding of participants, Blinding of outcome assessment, Incomplete outcome data, Selective reporting and other bias. Each domain classifies studies into low, unclear and high risk of bias. A third author (S.Y.L.) adjusted any disagreements by discussion.

### 2.5. Type of Interventions and Outcomes

We defined high intraoperative inspired oxygen concentration as fraction of inspired oxygen (FiO_2_) ≥ 0.8 and low concentration oxygen as FiO_2_ ≤ 0.5 during surgery or emergence. Partial pressure of oxygen (PaO_2_) was considered as primary outcome. The secondary outcomes included alveolar-arterial oxygen gradient (AaDO_2_), severity of atelectasis (surface of atelectasis to total lung surface observed on computed tomography) and blood oxygen saturation (SpO_2_). Atelectasis was defined as an area with density between –100 and +100 Hounsfield unit. 

### 2.6. Data Synthesis and Statistical Analyses (Meta-Analysis)

Revman 5.3 software (Cochrane Collaboration, Oxford, UK) and Comprehensive Meta-Analysis 3.3.070 software (CMA, Biostat, Englewood, NJ, USA) were used for data synthesis and analysis. Since all outcomes in this study were continuous variables, we calculated mean difference (MD) and 95% confidence intervals (CI) and presented our findings as forest plot with 95% CIs using random effects model only. In case of the results were presented in median and ranges, we used Wan’s formula to estimate means and SDs [15]. 

### 2.7. Assessment of Heterogeneity

The degree of heterogeneity among the studies was quantified by I^2^ statistic. We calculated I^2^ and suggested range for low, moderate, and high as 0–50%, 50–75%, and 75–100%. According to assumption that airway device will result in heterogeneous outcomes, subgroup analysis was performed depending on airway device (endotracheal tube vs. laryngeal mask airway).

## 3. Results

### 3.1. Characteristic of Trials and Patients

**Figure 1:**
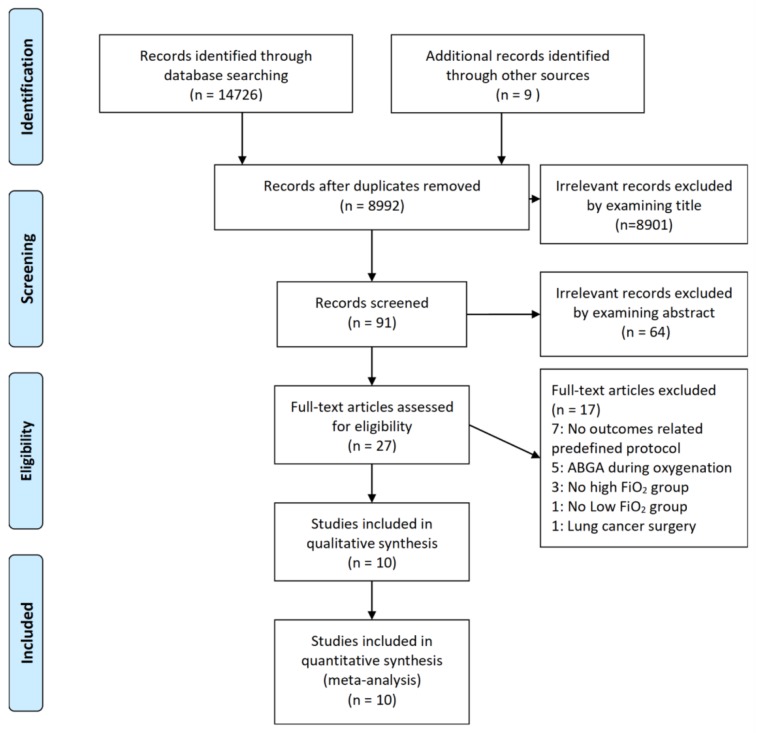
Flow diagram of included and excluded studies.

A total of 14,726 records published up to August 2018 were found through database searching. Nine studies used in previous meta-analysis evaluating pulmonary outcomes were also included [16]. On selection, 5743 records were excluded due to duplicated reports and 8901 records and 64 records were excluded by screening titles and abstracts, respectively. After evaluating full-text articles, 17 records were excluded because they reported no outcomes related to this study (*n* = 7), performed arterial blood gas analysis during intervention (*n* = 5), did not compare between high FiO_2_ and low FiO_2_ (*n* = 4) or included lung cancer surgery (*n* = 1). Finally, a total of 10 RCTs [17,18,19,20,21,22,23,24,25,26], with 787 patients, were included in final analysis (Figure 1). Among them, 391 patients were assigned to high FiO_2_ group and 396 patients to low FiO_2_ group. Details of included trials and patient characteristics are shown in Table 1.

### 3.2. Methodological Quality and Risk of Bias

**Figure 2:**
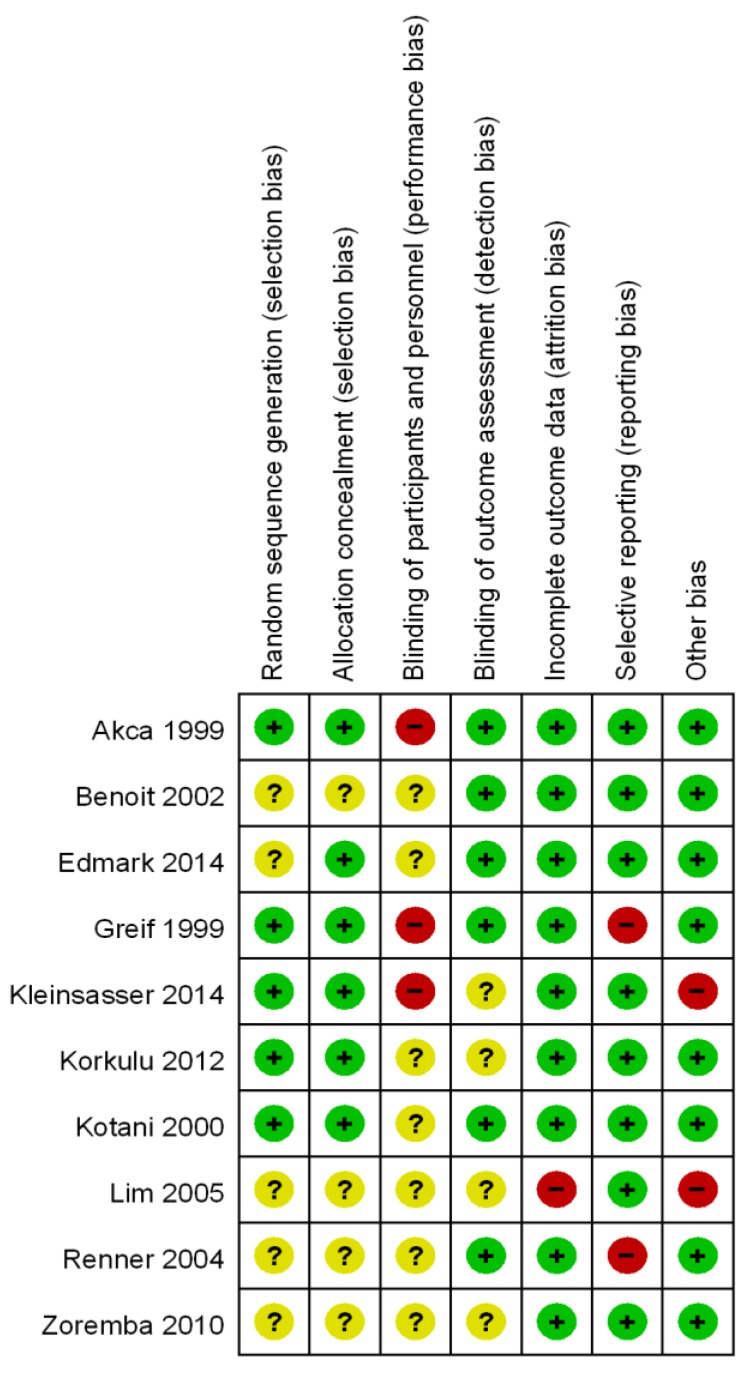
Risk of bias. Review author’s judgement about each risk of bias item for each included study.

Methodological quality and risk of bias are summarized in Figure 2. Patients were randomly allocated to each group in all studies, but there were no specific methods to generate randomization in half of the studies (5/10). Adequate allocation concealment is shown in 6/10 studies. The risk of performance bias was mostly unclear (7/10) or high (3/10), while the risk of detection bias was low (6/10) or unclear (4/10). It may be dangerous and impossible to blind participated anesthesiologists completely because of ventilator care and patient safety. Most trials were graded low risk of attrition bias (9/10), reporting bias (8/10) and other bias (8/10). Reasons for judgement of each bias were explicated in Table S2. 

### 3.3. Outcome Synthesis

**Figure 3:**
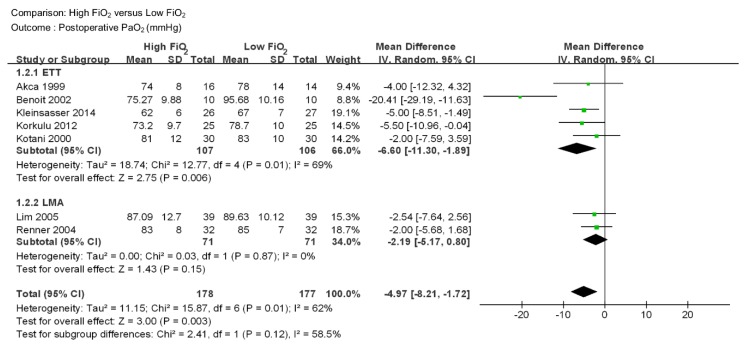
Forest plot for postoperative PaO_2_ (mmHg) in patients with high intraoperative inspired oxygen concentration versus low intraoperative inspired oxygen concentration. ETT = Endotracheal tube, LMA = Laryngeal Mask airway, CI = confidence interval.

**PaO_2_** PaO_2_ was reported in 7 studies including 355 patients (Figure 3) [17,18,21,22,23,24,25]. PaO_2_ was lower in high FiO_2_ group than low FiO_2_ group (MD −4.97 mmHg, 95% CI −8.21 to −1.72, *p* = 0.003). A moderate level of heterogeneity among the studies was observed (I^2^ = 62%, *p* = 0.01). In subgroup analysis, there were 5 studies in the endotracheal tube group [17,18,21,22,23] and 2 studies in the laryngeal mask airway group [24,25]. Each group included 213 and 142 patients, respectively. PaO_2_ was lower in high FiO_2_ in the endotracheal tube group (MD −6.60 mmHg, 95% CI –22.30 to –1.89, *p* = 0.006), while no significant difference was found in the laryngeal mask airway group (MD -2.19 mmHg, 95% CI -5.17 to 0.80, *p* = 0.15). The level of heterogeneity was moderate for the endotracheal tube group (I^2^=69%, *p* = 0.01) and low for the laryngeal mask airway group (I^2^=0%, *p* = 0.87).

**Figure 4:**
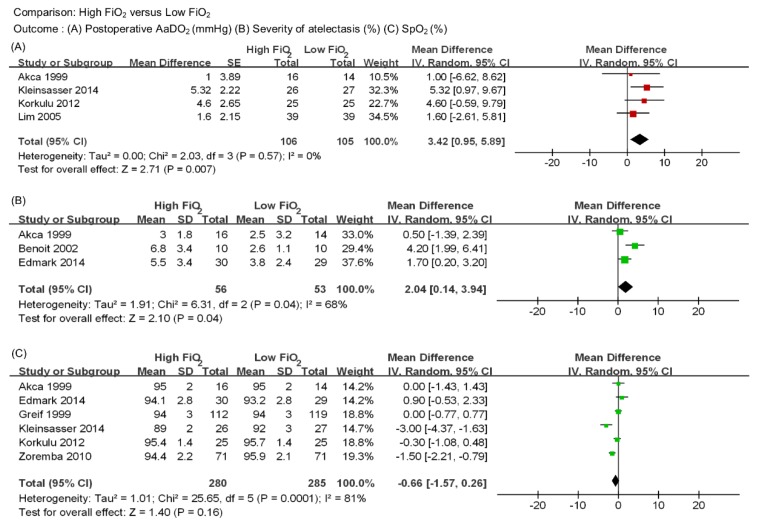
Forest plot for (**A**) postoperative AaDO_2_ (mmHg), (**B**) severity of atelectasis (%), (**C**) postoperative SpO_2_ (%).

**AaDO_2_** AaDO_2_ was reported in 4 studies including 211 patients (Figure 4A) [17,21,22,24]. AaDO_2_ was significantly higher in the high FiO_2_ group than the low FiO_2_ group (MD 3.42 mmHg, 95% CI 0.95 to 5.89, *p* = 0.007). A low level of heterogeneity was observed (I^2^ = 0, *p* = 0.57). Subgroup analysis was considered inappropriate because of the single study in the laryngeal mask airway group.

**Severity of atelectasis** Severity of atelectasis was reported in 3 studies including 109 patients (Figure 4B) [17,18,19]. As mentioned above, severity was measured by extent of atelectasis surface and expressed as a percentage of total lung surfaces. Relatively large amounts of atelectasis were observed in the high FiO_2_ group than the low FiO_2_ group (MD 2.04%, 95% CI 0.14 to 3.94, *p* = 0.04) with a moderate level of heterogeneity (I^2^ =68%, *p* = 0.04).

**SpO_2_** SpO_2_ was reported in 6 studies including 565 patients (Figure 4C) [17,19,20,21,22,26]. There were no significant differences in SpO_2_ during recovery between two groups (MD −0.66%, 95% CI −1.57 to 0.26, *p* = 0.16). A high level of heterogeneity existed among the studies (I^2^ = 81%, *p* = 0.0001).

## 4. Discussion

This meta-analysis included 10 randomized controlled trials—a total of 787 patients—assessing the postoperative pulmonary parameters according to the intraoperative inspired oxygen fraction in patients with general anesthesia. This meta-analysis suggests that high intraoperative inspired oxygen fraction decreased PaO_2_ and increased both AaDO_2_ and the severity of atelectasis postoperatively compared with low intraoperative inspired oxygen fraction. However, no significant difference in postoperative SpO_2_ was observed between the two groups.

Postoperative PaO_2_ was significantly lower in the high FiO_2_ group compared with the low FiO_2_ group. In general, postoperative hypoxemia resulted from hypoventilation, atelectasis, ventilation-perfusion mismatch, central nervous system depression or inadequate antagonism of neuromuscular blockade after anesthesia [27]. This finding may be due to increased shunt caused by absorption atelectasis with high inspired oxygen fraction [10].

AaDO_2_ is an indicator of pulmonary gas exchange with a normal value of about <20 mmHg in room air condition [28]. AaDO_2_ can also be a useful indicator in evaluating the shunt since it may be augmented by a shunt, ventilation-perfusion mismatch, or defect in diffusion [29]. The present meta-analysis suggested that postoperative AaDO_2_ is significantly higher in the high FiO_2_ group compared with the low FiO_2_ group. It also showed that high intraoperative inspired oxygen fraction increased the shunt with absorption atelectasis and impaired gas exchange postoperatively.

For this study, the severity of atelectasis was measured by comparing the extent of atelectasis to total lung surface. Atelectasis was found to be more severe in the high FiO_2_ group (5.0 ± 3.6%) compared with the low FiO_2_ group (3.2 ± 2.5%). Event short-term use of pure oxygen may increase absorptive atelectasis and the area of atelectasis is correlated with the degree of pulmonary shunt [9]. In the current study, high intraoperative inspired oxygen fraction may be considered to produce more atelectasis and, consequently, this also may be related to lower PaO_2_ and higher AaDO_2_ in the high FiO_2_ group.

SpO_2_ was comparable between two groups despite significant differences in other outcomes in the present analysis. Pulse oximetry is a standard of care during anesthesia monitoring. The oxyhemoglobin saturation curve has S shape and, in the steep part of the oxyhemoglobin saturation curve, a small change in oxygen partial pressure leads to an abrupt drop in saturation [21]. However, SpO_2_ may mask existing oxygenation defects in ventilated patients with higher than normal oxygen fractions [30]. Therefore, although less invasive, pulse oximetry may underestimate the severity of hypoxemia when compared with the invasive arterial blood gas analysis (ABGA) [31].

There are a few reasons for heterogeneity. Subgroup analysis revealed that postoperative PaO_2_ was significantly lower in the high FiO_2_ group compared with the low FiO_2_ group with the use of an endotracheal tube, whereas there was no significant difference in PaO_2_ between the two groups when the laryngeal mask airway was used. An endotracheal tube may stimulate the trachea and provoke bronchoconstriction, which seems to reduce PaO_2_ in the postoperative period [32]. Additionally, with an endotracheal tube, mucociliary function may be weakened [32], and accumulating secretions can result in atelectasis and shunt during anesthesia [33]. Another reason for the heterogeneity may be attributable to ABGA being performed at different times in each study. Blood samples for ABGA were drawn at 30 min [18,24,25] or 60 min [21,25] after extubation in some studies, whereas in other studies, it was performed on the first postoperative day [17,22,23].

There are limitations in this meta-analysis. First, there are significant differences in the short-term pulmonary parameters, such as PaO_2_ and AaDO_2_, according to intraoperative inspired oxygen saturation and further studies on long-term clinical prognosis are needed. Second, in addition to inspired oxygen concentration, there are numerous factors affecting postoperative pulmonary parameters such as intraoperative protective ventilation and neuromuscular blockade [1]. In the current study, the funnel plot seems to be asymmetrical (Supplemental Figure S1) and this phenomenon may be due to the methodological differences among the studies [34]. Benoit et al applied recruit maneuver 10 min before the end of surgery [18], which may cause not only asymmetrical funnel plot but also heterogeneity. Third, this study analyzed the postoperative PaO_2_ which was measured after the termination of intervention. However, intraoperative PaO_2_ could have the potential to detect early pulmonary impairment factors such as atelectasis or compliance changes, which may influence the postoperative measures in relatively short procedures.

## 5. Conclusions

In conclusion, high inspired oxygen fraction during anesthesia would deteriorate postoperative pulmonary parameters, including postoperative partial pressure of oxygen, alveolar-arterial oxygen gradient, and severity of atelectasis; and therefore, cautious approach is required in determining the inspired oxygen concentration in patients susceptible to postoperative pulmonary complications.

## Figures and Tables

**Table 1:** Baseline characteristics and population of the included randomized trials (*n* = 10).

Author	Year	Design	No. of Patients (High FiO_2_/Low FiO_2_)	Language	Age		FiO_2_	Airway Device	Maintenance Anesthetics	Intervention Period	Type of Surgery
					High FiO_2_	Low FiO_2_					
Akca	1999	RCT	30 (16/14)	English	49 ± 14	41 ± 10	0.8/0.3	ETT	IA	Intraoperative and postoperative 2 hour	Colon resection
Benoit	2002	RCT	20 (10/10)	English	40 ± 14	32 ± 11	1.0/0.4	ETT	IV	Emergence	Surgery of the extremities
Edmark	2014	RCT	59 (30/29)	English	53 ± 12	53 ± 11	1.0/0.3	LMA	IV	Emergence	Orthopedic surgery
Greif	1999	RCT	231 (112/119)	English	59 ± 14	60 ± 13	0.8/0.3	ETT	IA	Intraoperative and postoperative 2 hour	Colon or rectum resection
Kleinsasser	2014	RCT	53 (26/27)	English	68 ± 13	65 ± 10	1.0/0.3	ETT	IV	Emergence	Carotid endarterectomy
Korkulu	2012	RCT	50 (25/25)	Turkish	41.7 ± 11.6	46.2 ± 11.3	1.0/0.4	ETT	IA	Intraoperative	Laparoscopic cholecystectomy
Kotani	2000	RCT	60 (30/30)	English	49 ± 8	48 ± 9	1.0/0.3	ETT	IV	Intraoperative	Orthopedic surgery
Lim	2005	RCT	78 (39/39)	Korean	43.5 ± 14.3	41.5 ± 15.1	1.0/0.3	LMA	IV or IA	Emergence	Surgery of extremities
Renner	2004	RCT	64 (32/32)	English	31 ± 7	30 ± 7	1.0/0.3	LMA	IV	Emergence	Peripheral musculoskeletal surgery
Zoremba	2010	RCT	142 (71/71)	English	51 ± 11	50 ± 12	0.8/0.4	ETT	IV	Intraoperative	Minor peripheral surgery

Age is expressed as the mean ± SD, RCT = randomized controlled trial, ETT = Endotracheal tube, LMA = Laryngeal mask airway, IV = Intravenous anesthetics, IA = Inhalational anesthetics.

## Supplementary Materials

The following are available online at https://www.mdpi.com/2077-0383/8/5/583/s1, Table S1: Search strategy for each database, Table S2: Details for judgement for each risk of bias for randomized controlled studies, Figure S1: Funnel plot of primary outcome.
