# Supplementary material for: The Effects of Intraoperative Inspired Oxygen Fraction on Postoperative Pulmonary Parameters in Patients with General Anesthesia: A Systemic Review and Meta-Analysis
Source: J Clin Med. 2019 Apr 28;8(5):583. doi: 10.3390/jcm8050583 (PMC6572026; doi:10.3390/jcm8050583)
Supplement: Supplementary file 1 [file jcm-08-00583-s001.zip › Table S1.docx]

Supplemental Table 1. Search strategy for each database.

| Database | Order | Keywords |
| --- | --- | --- |
| MEDLINE | #1 | Anesthesia[mh] |
|  | #2 | Anesth*[TIAB] |
|  | #3 | #1 OR #2 |
|  | #4 | “Postoperative period”[mh] |
|  | #5 | “Postoperative complications”[mh] |
|  | #6 | “Postoperative care”[mh] |
|  | #7 | Postoperative[TIAB] |
|  | #8 | #4 OR #5 OR #6 OR #7 |
|  | #9 | #3 AND #8 |
|  | #10 | “Airway extubation”[mh] |
|  | #11 | extubate*[TIAB] OR remov*[TIAB] OR emergence[TIAB] |
|  | #12 | #10 OR #11 |
|  | #13 | #3 AND #12 |
|  | #14 | #9 OR #13 |
|  | #15 | Oxygen[mh] |
|  | #16 | Hyperoxia[mh] |
|  | #17 | Hyperoxemia[TIAB] OR hyperoxic[TIAB] |
|  | #18 | “100% oxygen”[TIAB] OR “100% O2”[TIAB] |
|  | #19 | supplement* [TIAB] OR Fraction*[TIAB] OR insufflat*[TIAB] OR inspirat*[TIAB] inspired[TIAB] OR inhalat*[TIAB] OR administrat*[TIAB] OR concentrat*[TIAB] OR breath*[TIAB] |
|  | #20 | oxygen*[TIAB] OR O2[TIAB] |
|  | #21 | #19 AND #20 |
|  | #22 | #15 OR #16 OR #17 OR #18 OR #21 |
|  | #23 | #14 AND #22 |
|  | #24 | (randomized controlled trial [pt] OR controlled clinical trial [pt] OR randomized [tiab] OR placebo [tiab] OR drug therapy [sh] OR randomly [tiab] OR trial [tiab] OR groups [tiab]) NOT (animals [mh] NOT humans [mh]) |
|  | #25 | #23 AND #24  Total items : 1478 |
| EMBASE | #1 | Anesthesia/exp |
|  | #2 | anesth*:ab,ti |
|  | #3 | #1 OR #2 |
|  | #4 | ‘postoperative period’/de |
|  | #5 | ‘postoperative complication’/de |
|  | #6 | ‘postoperative care’/de |
|  | #7 | postoperative:ab,ti |
|  | #8 | #4 OR #5 OR #6 OR #7 |
|  | #9 | #3 AND #8 |
|  | #10 | extubation/de |
|  | #11 | extubat*:ab,ti OR remov*:ab,ti OR emergence:ab,ti |
|  | #12 | #10 OR #11 |
|  | #13 | #3 AND #12 |
|  | #14 | #9 OR #13 |
|  | #15 | oxygen/de |
|  | #16 | hyperoxia/de |
|  | #17 | hyperoxia:ab,ti OR hyperoxic:ab,ti |
|  | #18 | ‘100% oxygen’:ab,ti OR ‘100% O2’:ab,ti |
|  | #19 | supplement*:ab,ti OR fraction*:ab,ti OR insufflat*:ab,ti OR inspirat*:ab,ti OR inspired:ab,ti OR inhalat*:ab,ti OR administrat*:ab,ti OR concentrat*ab,ti OR breath*:ab,ti |
|  | #20 | oxygen*:ab,ti OR O2:ab,ti |
|  | #21 | #19 AND #20 |
|  | #22 | #15 OR #16 OR #17 OR #18 OR #21 |
|  | #23 | #14 AND #22 |
|  | #24 | 'crossover procedure'/exp OR 'crossover procedure' OR 'double blind procedure'/exp OR 'double blind procedure' OR 'randomized controlled trial'/exp OR 'randomized controlled trial' OR 'single blind procedure'/exp OR 'single blind procedure' OR random* OR factorial* OR crossover* OR 'cross over' OR 'cross-over' OR placebo* OR (doubl* AND blind*) OR (singl* AND blind*) OR assign* OR allocat* OR volunteer* |
|  | #25 | #23 AND #24 |
|  | #26 | #25 AND [embase]/lim |
|  |  | Total items : 1785 |
| CENTRAL | #1 | [mh anesthesia] |
|  | #2 | anesth*:ti,ab,kw |
|  | #3 | #1 OR #2 |
|  | #4 | [mh “postoperative period”] |
|  | #5 | [mh “postoperative complications”] |
|  | #6 | [mh “postoperative care”] |
|  | #7 | postoperative:ti,ab,kw |
|  | #8 | #4 OR #5 OR #6 OR #7 |
|  | #9 | #3 AND #8 |
|  | #10 | [mh “airway extubation”] |
|  | #11 | extubat*:ti,ab,kw OR remov*:ti,ab,kw OR emergence:ti,ab,kw |
|  | #12 | #10 OR #11 |
|  | #13 | #3 AND #12 |
|  | #14 | #9 OR #13 |
|  | #15 | [mh oxygen] |
|  | #16 | [mh hyperoxia] |
|  | #17 | hyperoxia:ti,ab,kw OR hyperoxic:ti,ab,kw |
|  | #18 | “100% oxgen”:ti,ab,kw OR “100% O2”:ti,ab,kw |
|  | #19 | supplement*:ti,ab,kw OR fraction*:ti,ab,kw OR insufflat*:ti,ab,kw OR inspirat*:ti,ab,kw OR inspired:ti,ab,kw OR inhalat*:ti,ab,kw OR administrat*:ti,ab,kw OR concentrat*ti,ab,kw OR breath*:ti,ab,kw |
|  | #20 | oxygen*:ti,ab,kw OR O2:ti,ab,kw |
|  | #21 | #19 AND #20 |
|  | #22 | #15 OR #16 OR #17 OR 18 OR #21 |
|  | #23 | #14 AND #22 |
|  | #24 | #23(in Trials) |
|  |  | Total items : 5735 |
| CINHAL | S1 | MH(anesthesia+) |
|  | S2 | TI(anesth*) OR AB(anesth*) |
|  | S3 | S1 OR S2 |
|  | S4 | MH(postoperative period) |
|  | S5 | MH(postoperative complications) |
|  | S6 | MH(postoperative care) |
|  | S7 | TI(postoperative) OR AB(postoperative) |
|  | S8 | S4 OR S5 OR S6 OR S7 |
|  | S9 | S3 AND S8 |
|  | S10 | MH(extubation) |
|  | S11 | TI(extubat* OR remov* OR emergence) OR AB(extubat* OR remov* OR emergence) |
|  | S12 | S10 OR S11 |
|  | S13 | S3 AND S12 |
|  | S14 | S9 OR S13 |
|  | S15 | MH(oxygen) |
|  | S16 | MH(hyperoxia) |
|  | S17 | TI(hyperoxia OR hyperoxic) OR AB(hyperoxia OR hyperoxic) |
|  | S18 | TI(100% oxygen OR 100% O2) OR AB(100% oxygen OR 100% O2) |
|  | S19 | TI(supplement* OR fraction* OR insufflat* OR inspirat* OR inspired OR inhalat* OR administrat* OR concentrat* OR breath*) OR AB(supplement* OR fraction* OR insufflat* OR inspirat* OR inspired OR inhalat* OR administrat* OR concentrat* OR breath*) |
|  | S20 | TI(oxygen* OR O2) OR AB(oxygen* OR O2) |
|  | S21 | S19 AND S20 |
|  | S22 | S15 OR S16 OR S17 OR S18 OR S21 |
|  | S23 | S14 AND S22 |
|  | S24 | (MH "Clinical Trials+") OR (PT Clinical trial) OR (TX clinic* n1 trial*) OR TX ( (singl* n1 blind*) OR (singl* n1 mask*) ) OR TX ( (doubl* n1 blind*) OR (doubl* n1 mask*) ) OR TX ( (tripl* n1 blind*) OR (tripl* n1 mask*) ) OR TX ( (trebl* n1 blind*) or (trebl* n1 mask*) ) OR TX randomi* control* trial* OR (MH "Random Assignment") OR TX random* allocat* OR TX placebo* OR (MH "Placebos") OR (MH "Quantitative Studies") OR TX allocat* random* |
|  | S25 | S23 AND S24 |
| SCOPUS | #1 | INDEXTERMS(anesthesia) |
|  | #2 | TITLE-ABS(anesth*) |
|  | #3 | #1 OR #2 |
|  | #4 | INDEXTERMS(postoperative period) |
|  | #5 | INDEXTERMS(postoperative complications) |
|  | #6 | INDEXTERMS(postoperative care) |
|  | #7 | TITLE-ABS(postoperative) |
|  | #8 | #4 OR #5 OR #6 OR #7 |
|  | #9 | #3 AND #8 |
|  | #10 | INDEXTERMS(airway extubation) |
|  | #11 | TITLE-ABS(extubat*) OR TITLE-ABS(remov*) OR TITLE-ABS(emergence) |
|  | #12 | #10 OR #11 |
|  | #13 | #3 AND #12 |
|  | #14 | #9 OR #13 |
|  | #15 | INDEXTERMS(oxygen) |
|  | #16 | INDEXTERMS(hyperoxia) |
|  | #17 | TITLE-ABS(hyperoxia) OR TITLE-ABS(hyperoxic) |
|  | #18 | TITLE-ABS(100% oxygen) OR TITLE-ABS(100% O2) |
|  | #19 | TITLE-ABS(supplement*) OR TITLE-ABS(fraction*) OR TITLE-ABS(insufflat*) OR TITLE-ABS(inspirat*) OR TITLE-ABS(inspired) OR TITLE-ABS(inhalat*) OR TITLE-ABS(administrat*) OR TITLE-ABS(concentrat*) OR TITLE-ABS(breath*) |
|  | #20 | TITLE-ABS(oxygen*) OR TITLE-ABS(O2) |
|  | #21 | #19 AND #20 |
|  | #22 | #15 OR #16 OR #17 OR #18 OR #21 |
|  | #23 | #14 AND #22 |
|  | #24 | (INDEXTERMS(randomized controlled trial) OR INDEXTERMS(controlled clinical trial) OR TITLE-ABS(randomized) OR TITLE-ABS(placebo) OR INDEXTERMS(drug therapy) OR TITLE-ABS(randomly) OR TITLE-ABS(trial) OR TITLE-ABS(groups)) AND NOT (INDEXTERMS(animals) AND NOT INDEXTERMS(humans)) |
|  | #25 | #23 AND #24 |
|  |  | Total items : 4740 |
| Web of Science | #1 | anesth* |
|  | #2 | postoperative |
|  | #3 | #1 AND #2 |
|  | #4 | extubat* OR remov* OR emergence |
|  | #5 | #1 AND #4 |
|  | #6 | #3 OR #5 |
|  | #7 | oxygen* OR O2 |
|  | #8 | hyperoxia OR hyperoxemia OR hyperoxic |
|  | #9 | 100% oxygen OR 100% O2 |
|  | #10 | supplement* OR fraction* OR insufflat* OR inspirat* OR inspired OR inhalat* administrat* OR concentrat* OR breath* |
|  | #11 | #7 AND #10 |
|  | #12 | #8 OR #9 OR #11 |
|  | #13 | #6 AND #12 |
|  | #14 | (clinical trial* OR research design OR comparative stud* OR evaluation stud* OR controlled trial* OR follow-up stud* OR prospective stud* OR random* OR placebo* OR “single blind*” OR double blind*) |
|  | #15 | #13 AND #14 |
|  |  | Total items : 929 |
