# Supplementary material for: The Effects of Intraoperative Inspired Oxygen Fraction on Postoperative Pulmonary Parameters in Patients with General Anesthesia: A Systemic Review and Meta-Analysis
Source: J Clin Med. 2019 Apr 28;8(5):583. doi: 10.3390/jcm8050583 (PMC6572026; doi:10.3390/jcm8050583)
Supplement: Supplementary file 1 [file jcm-08-00583-s001.zip › Table S2.docx]

Supplementary Table 3. Details for judgement for each risk of bias for randomized controlled studies.

| Study | Bias | Author’s judgement | Reason for judgement |
| --- | --- | --- | --- |
| Akca 1999 | Random sequence generation  (selection bias) | Low | using a reproducible set of computer-generated random numbers. |
|  | Allocation concealment  (selection bias) | Low | The assignments were kept in sealed, sequentially numbered envelopes until used. |
|  | Blinding  (performance) | High | The anesthesiologists and investigator assigned to perioperative management were aware of the group assignment. |
|  | Blinding  (detection bias) | Low | Patients, nurses, surgeons and other investigators were blinded to the group assignments. To maintain blinding, the perioperative blood gas results were not recorded on the postanesthesia care notes or elsewhere in patient records. |
|  | Incomplete outcome data  (attrition bias) | Low | Outcomes were reported for 30 patients. |
|  | Selective reporting  (reporting bias) | Low | All pre-specified and expected outcomes are reported |
|  | Other bias | Low | No other bias was detected. |
| Benoit 2002 | Random sequence generation  (selection bias) | Unclear | There is no description of method used for random sequence generation. |
|  | Allocation concealment  (selection bias) | Unclear | There is no description of allocation concealment |
|  | Blinding  (performance bias) | Unclear | There is no description. |
|  | Blinding  (detection bias) | Low | Only one investigator performed these measurements, and he was blinded to the randomization. |
|  | Incomplete outcome data  (attrition bias) | Low | Outcomes were reported for all patients. |
|  | Selective reporting  (reporting bias) | Low | All pre-specified and expected outcomes are reported. |
|  | Other bias | Low | No other bias was detected. |
| Edmark 2014 | Random sequence generation  (selection bias) | Unclear | There is no description of method used for random sequence generation. |
|  | Allocation concealment  (selection bias) | Low | Using the sealed envelope technique. |
|  | Blinding  (performance bias) | Unclear | There is no description. |
|  | Blinding  (detection bias) | Low | A radiologist, blinded to group affiliation of the patients, measured the area of atelectasis. |
|  | Incomplete outcome data  (attrition bias) | Low | Outcomes were reported for 59 patients. |
|  | Selective reporting  (reporting bias) | Low | All pre-specified and expected outcomes are reported. |
|  | Other bias | Low | No other bias was detected |
| Greif 1999 | Random sequence generation  (selection bias) | Low | The treatment randomization was based on computer-generated codes |
|  | Allocation concealment  (selection bias) | Low | Maintained in sealed, opaque envelopes until just after induction of anesthesia. |
|  | Blinding  (performance bias) | High | The anesthesiologists was aware of the administered oxygen concentration. |
|  | Blinding  (detection bias) | Low | Patients and surgeons and the nurses reporting postoperative nausea and vomiting were blinded to group assignments and actual inspired oxygen concentration. |
|  | Incomplete outcome data  (attrition bias) | Low | Outcomes were reported for 231 patients. |
|  | Selective reporting  (reporting bias) | High | Blood gas analysis was not reported. |
|  | Other bias | Low | No other bias was detected. |
| Kleinsasser 2014 | Random sequence generation  (selection bias) | Low | Randomization was performed by using Research Randomizer, a long standing on-line tool. |
|  | Allocation concealment  (selection bias) | Low | Using opaque envelopes labeled with the sequential number |
|  | Blinding  (performance bias) | High | The anesthetist was not blinded in terms of group allocation |
|  | Blinding  (detection bias) | Unclear | There is no description. |
|  | Incomplete outcome data  (attrition bias) | Low | Outcomes were reported for all patients. |
|  | Selective reporting  (reporting bias) | Low | All pre-specified and expected outcomes are reported. |
|  | Other bias | High | We examined predominantly men, failure to perform neasurements later than 60 min after extubation. |
| Korkulu 2012 | Random sequence generation  (selection bias) | Low | The randomization method was obtained by a lot from an envelope with an equal number of papers |
|  | Allocation concealment  (selection bias) | Low | The randomization method was obtained by a lot from an envelope with an equal number of papers |
|  | Blinding  (performance bias) | Unclear | There is no description. |
|  | Blinding  (detection bias) | Unclear | There is no description. |
|  | Incomplete outcome data  (attrition bias) | Low | Outcomes were reported for all patients. |
|  | Selective reporting  (reporting bias) | Low | All pre-specified and expected outcomes are reported. |
|  | Other bias | Low | No other bias was detected. |
| Kotani 2000 | Random sequence generation  (selection bias) | Low | The computer-generated assignments |
|  | Allocation concealment  (selection bias) | Low | Kept in sealed, sequentially numbered envelops until use |
|  | Blinding  (performance bias) | Unclear | There is no description. |
|  | Blinding  (detection bias) | Low | A physician who was unware of the patient’s group assignment and intraoperative treatment. |
|  | Incomplete outcome data  (attrition bias) | Low | Outcomes were reported for 60 patients |
|  | Selective reporting  (reporting bias) | Low | All pre-specified and expected outcomes are reported. |
|  | Other bias | Low | No other bias was detected. |
| Lim 2005 | Random sequence generation  (selection bias) | Unclear | There is no description of method used for random sequence generation. |
|  | Allocation concealment  (selection bias) | Unclear | There is no description. |
|  | Blinding  (performance bias) | Unclear | There is no description. |
|  | Blinding  (detection bias) | Unclear | There is no description. |
|  | Incomplete outcome data  (attrition bias) | High | There are differences between analyzed patients (n=77) and reported patients(n=78), |
|  | Selective reporting  (reporting bias) | Low | All pre-specified and expected outcomes are reported. |
|  | Other bias | High | There was no consensus for the criteria of LMA removal among 4 anesthesiologists. |
| Renner 2004 | Random sequence generation  (selection bias) | Unclear | There is no description of method used for random sequence generation. |
|  | Allocation concealment  (selection bias) | Unclear | There is no description. |
|  | Blinding  (performance bias) | Unclear | There is no description. |
|  | Blinding  (detection bias) | Low | Intraoperative and postoperative data were collected by a blinded investigator. |
|  | Incomplete outcome data  (attrition bias) | Low | Outcomes were reported for 64 patients. |
|  | Selective reporting  (reporting bias) | High | Predefined intraoperative complications were not reported |
|  | Other bias | Low | No other bias was detected. |
| Zoremba 2010 | Random sequence generation  (selection bias) | Unclear | There is no description of method used for random sequence generation. |
|  | Allocation concealment  (selection bias) | Unclear | There is no description. |
|  | Blinding  (performance bias) | Unclear | There is no description. |
|  | Blinding  (detection bias) | Unclear | There is no description. |
|  | Incomplete outcome data  (attrition bias) | Low | Outcomes were reported for all patients. |
|  | Selective reporting  (reporting bias) | Low | All pre-specified and expected outcomes are reported. |
|  | Other bias | Low | No other bias was detected. |
