# Supplementary figures and images for: The Effects of Intraoperative Inspired Oxygen Fraction on Postoperative Pulmonary Parameters in Patients with General Anesthesia: A Systemic Review and Meta-Analysis
Source: J Clin Med. 2019 Apr 28;8(5):583. doi: 10.3390/jcm8050583 (PMC6572026; doi:10.3390/jcm8050583)

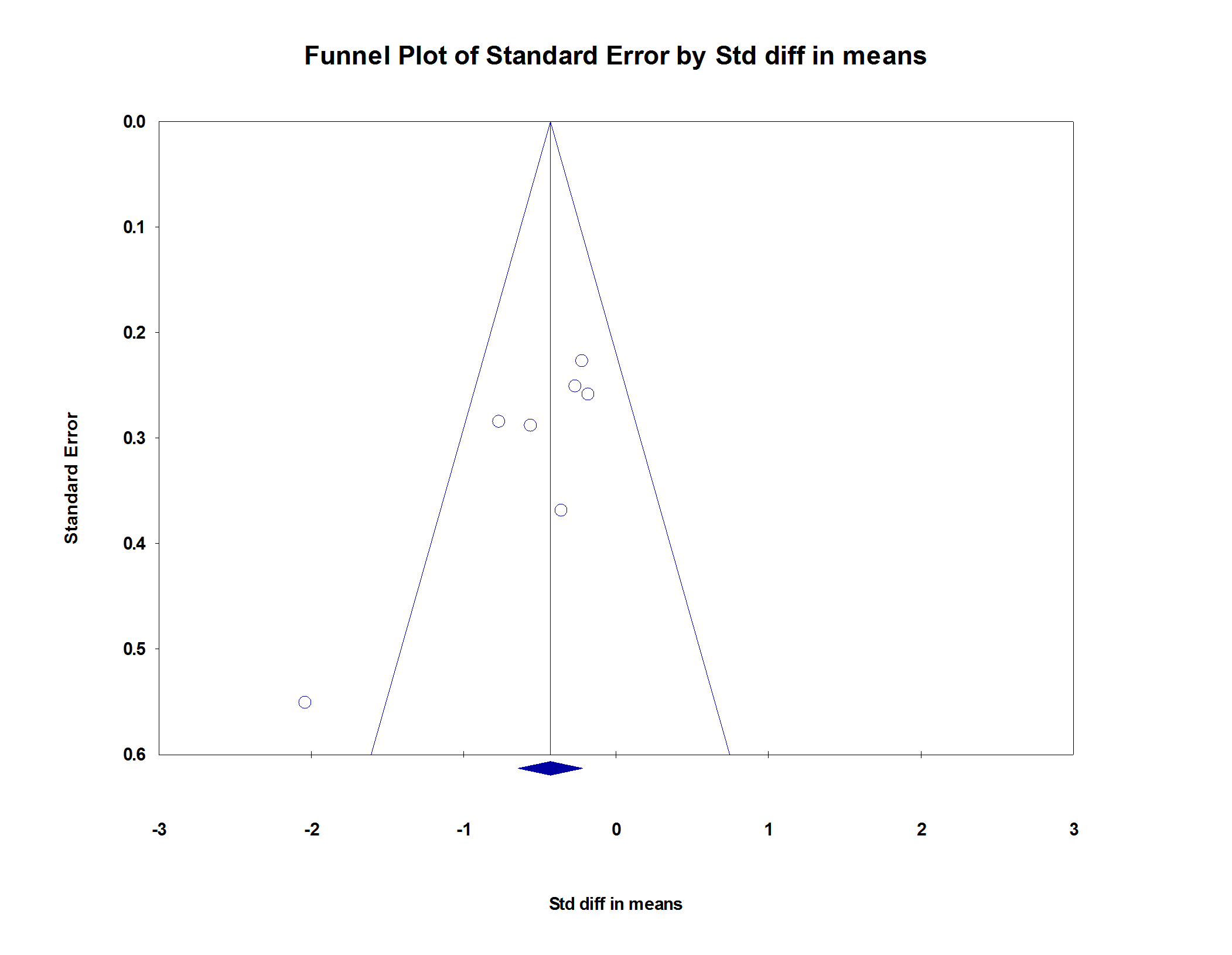

Supplement: Supplementary file 1 [file jcm-08-00583-s001.zip › Supplemental Figure S1.tif]
